# Feasibility of a new method using two-dimensional transesophageal echocardiography for aortic annular sizing in patients undergoing transcatheter aortic valve implantation; a case–control study

**DOI:** 10.1186/s12872-015-0072-7

**Published:** 2015-07-28

**Authors:** Mohammad A. Sherif, Joerg Herold, Wolfram Voelker, Octavian Maniuc, Georg Ertl, Christian Praast, Ruediger Christian Braun-Dullaeus

**Affiliations:** Cardiology Department, Wuerzburg University Clinic, Oberduerrbacher Street 6, 97080 Wuerzburg, Germany; Cardiology Department, Magdeburg University Clinic, Magdeburg, Germany

**Keywords:** TAVI, Sizing, Echocardiography

## Abstract

**Background:**

Accurate preoperative assessment of the aortic annulus dimension is crucial for successful transcatheter aortic valve implantation (TAVI). In this study we validated a new method using two-dimensional transesophageal echocardiography (2D-TEE) for measurement of the aortic annulus prior to TAVI.

**Methods:**

We analysed 124 patients who underwent successful TAVI using a self-expandable prosthesis, divided equally into two groups; in the study group we used the cross sectional short axis 2D-TEE for measurement of the aortic annulus and in the control group we used the long axis 2D-TEE.

**Results:**

Both groups were comparable regarding the clinical parameters. On the other hand, patients in the study group had less left ventricular ejection fraction (38.9 % versus 45.6 %, *p* = 0.01). The aortic valve annulus was, although not statistically significant, smaller in the study group (21.58 versus 23.28 mm, *p* = 0.25).

Post procedural quantification of the aortic regurgitation revealed that only one patient in both groups had severe aortic regurgitation (AR), in this patient the valve was implanted deep. The incidence of significant AR was higher in the control group (29.0 % versus 12.9 %, *p* = 0.027).

**Conclusions:**

Sizing of the aortic valve annulus using cross-sectional 2D-TEE offers a safe and plausible method for patients undergoing TAVI using the self-expandable prosthesis and is significantly superior to using long axis 2D-TEE.

## Background

Accurate preoperative assessment of the aortic annulus dimension is crucial for successful transcatheter aortic valve implantation (TAVI). Under-sizing may result in prosthesis migration and severe paravalvular leakage [1]. In contrast, over-sizing may result in aortic annular rupture [2].

The choice of the prosthesis size was based only on two-dimensional transesophageal echocardiography (TEE) measurements. However, Recent research demonstrates that 2D-TEE may underestimates the annulus [3]. For this reason, alternative sizing methods based on multidetector computed tomography (MDCT) [4] und Three-dimensional (3D) TEE [5] have been developed.

MDCT has become the “gold standard” for non-invasive preoperative evaluation of the aortic root and aortic annulus prior to TAVI using the balloon expandable Edwards-Sapine bioprothesis (Edwards Sapien/Sapien XT, Edwards Lifesciences, Irvine, California) [6–8]. However, because of renal dysfunction in a population with a significant burden of comorbidities, MDCT is often not an option.

To the best of our knowledge, this is the first study aimed at comparing multiple methods of annulus measurements using 2D-TEE and its impact on the outcome after TAVI using the Medtronic CoreValve bioprosthesis (Medtronic, Inc. of Minneapolis, Minnesota).

In this study we examined a new method using 2D-TEE for non-invasive preoperative evaluation of the aortic annulus prior to TAVI using Medtronic CoreValve bioprosthesis.

## Methods

### Study design and patients

One hundred and twenty-four patients with severe symptomatic AS (AVA < 1 cm^2^ or AVA indexed to body surface area < 0.6 cm^2^/m^2^) were included in this study. TAVI was done using the Medtronic CoreValve bioprosthesis via transfemoral route in 2 centres. Clinical & anatomical selection criteria and device size selection were in line with the published investigational study for the third generation (18 F) CoreValve device. Description of the device and technical aspects of the procedure have been previously published [9]. We analysed 124 patients divided equally into two groups; the study group and the control group, each consisted of 62 patients.

This study has been performed in accordance with the ethical standards laid down in the 1964 Declaration of Helsinki and its later amendments. Because of the retrospective nature of the study, an ethical approval was not required for this study.

### Echocardiographic assessment

In both groups, a comprehensive TTE and TEE was performed preoperatively. The severity of aortic stenosis was assessed by the transvalvular mean gradient and aortic valve area (AVA), which was calculated with the continuity equation and planimetry. AR was quantified using color-flow techniques that included measurement of the width and area of the AR jet at the junction of the left ventricular outflow tract (LVOT) and the aortic annulus in the parasternal long axis view in relation to the maximum width and area of the LVOT at the same location [10].

Post-operatively, the presence, degree and type (paravalvular versus transvalvular) of AR were recorded in all patients using TTE and quantified according to the VARC criteria [11].

**Fig. 1:**
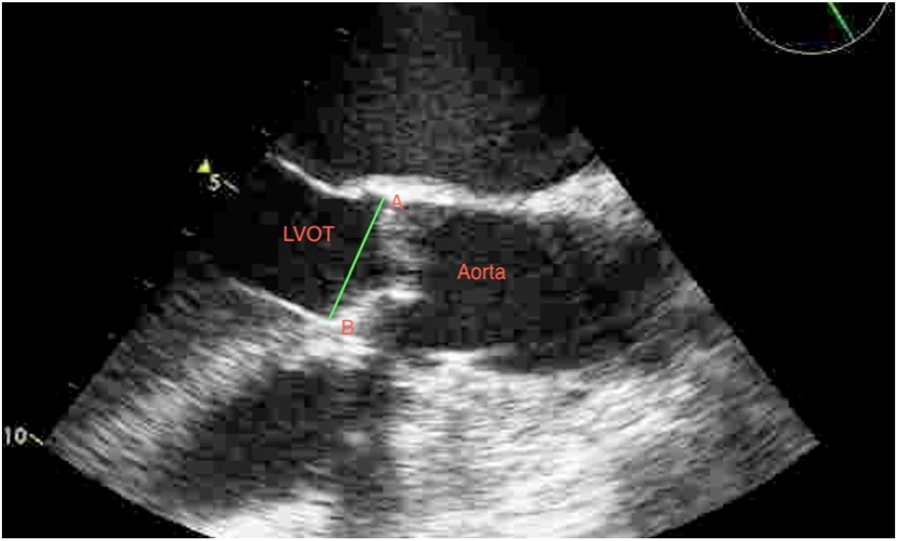
Measurement of the aortic annulus using TEE on the mid-oesophageal long-axis view of the ascending aorta and aortic valve at early-systole

In the control group the aortic annulus was measured as the distance between the insertion of two adjacent leaflets on the parasternal long axis view and on the mid-oesophageal long-axis view of the ascending aorta and aortic valve at early-systole [12]. Annular size measurement was performed using the enlarged view of the mid-oesophageal long axis (Approximately 110° to 140°, referred to as the “3-chamber view”) during the early systolic phase of the cardiac cycle. In this projection, the left ventricular chamber, outflow tract, and ascending aorta should be aligned along their long axes to ensure that the sagittal plane bisects the maximal diameter of the annulus. The aortic valve annulus was measured following the trailing edge–to–leading edge rule. The measurement was done from the hinge point of the right coronary cusp perpendicular to the long axis of the aorta (Fig. 1).

Measurement of the aortic annulus was used to determine the appropriate valve size.

**Fig. 2:**
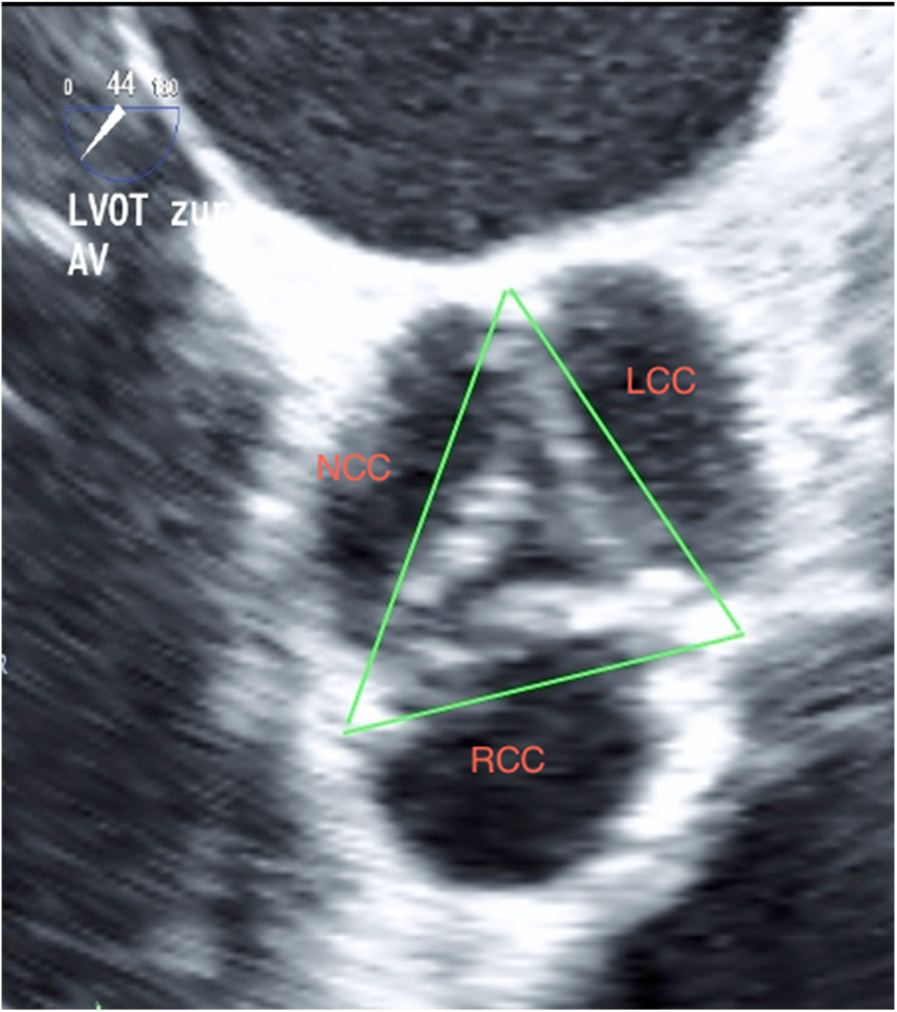
Annular size measurement using the enlarged view of the mid-oesophageal short axis (approximately 30° to 50°). The short-axis views of the aortic valve were generated at the insertion of the cusps in systole. LCC: left coronary cusp, RCC: right coronary cusp, NCC: non-coronary cusp

In the study group, annular size measurement was performed using the enlarged view of the mid-oesophageal short axis (approximately 30° to 50°) during the early systolic phase of the cardiac cycle. The short-axis views of the aortic valve were generated at the insertion of the cusps in systole. The mid-oesophageal AV short axis view (Fig. 2) was obtained from the mid-oesophageal window by advancing or withdrawing the probe until the AV comes into view and then turning the probe to centre the AV in the display. The image depth was adjusted to between 10 and 12 cm to position the AV in the middle of the display. Next, the multiplane angle was rotated forward to approximately 30 to 60° until a symmetrical image of all three cusps of the aortic valve and the coronary sinuses comes into view.

This view shows how the leaflets join together along trifoliate zones of apposition extending from peripheral attachments at the sinutubular junction to the centroid of the valvar orifice. These zones of apposition are the true commissures. The aortal end of the commissures correlates anatomically to the upper end of the interleaflet triangle and at the same time they represent the sinutubular junction (Fig. 2). Three lines were drawn between these points and the longest diameter was used for the purpose of sizing (Fig. 2).

### Statistical analysis

Statistical analysis was performed using the statistical package for social sciences (SPSS 20), IBM Corporation, New York, USA. The data of metric are given as mean and standard deviation. For the univariate analysis, the mean values and proportions of baseline variables were compared using Pearson’s *X*2 test. Reference to statistical significance was made by presenting a p value for a risk of 5 %. Comparisons of baseline and procedure-related characteristics of patients according were performed using *t*-test or Fishers exact test as appropriate.

## Results

Baseline clinical, hemodynamic, and procedural characteristics are shown in Table 1. Both groups were comparable regarding the clinical parameters. The aortic valve annulus was, although not statistically significant, smaller in the study group (21.58 versus 23.28 mm, *p* = 0.25). The study group had a worse ejection fraction at baseline (38.9 % versus 45.6 %, *p* = 0.01) (Table 1).

**Table 1:** Clinical and procedural characteristics for 124 patients who underwent TAVI

	Control group (n. 62)	Study group (n.62)	*p*-Value
Age, years	79.92 ± 7.79	79.61 ± 5.46	0.93
Body mass index	26.9 ± 5.32	28.3 ± 5.49	0.98
Female gender	53.2 %	64.5 %	0.21
Rhythm			
Sinus	66.1 %	61.3 %	0.56
Atrial fibrillation	25.8 %	35.5 %	0.24
Pace maker	8.1 %	3.2 %	0.22
Coronary Heart Disease	72.6 %	71.7 %	0.9
Peripheral artery disease	36.5 %	21 %	0.06
Previous myocardial infarction	17.5 %	12.9 %	0.39
Pre-Aortic regurgitation			
No	27.0 %	27.9 %	0.9
Mild	63.5 %	47.9 %	0.09
Moderate	9.5 %	24.2 %	0.02
Valve size			
26 mm	42 % (26)	48.4 % (30)	0.5
29 mm	51.6 % (32)	46.7 % (29)	0.6
31 mm	3.3 % (4)	4.9 % (3)	0.6
Mitral regurgitation			
No	9.5 %	19.7 %	0.11
Mild	71.4 %	52.5 %	0.03
Moderate	17.5 %	26.2 %	0.22
Severe	1.6 %	1.6 %	1
EF, %	45.6 ± 15.89	38.9 ± 12.77	0.01
Aortic valve area, cm2	0.7 ± 0.18	0.68 ± 0.17	0.57
Peak pressure gradient, mmHg	70.19 ± 25.15	71.54 ± 25.3	0.81
Mean pressure gradient, mmHg	45.9 ± 17.67	45.69 ± 17.77	0.94
Annulus, mm	23.28 ± 1.7	21.58 ± 1.6	0.25

*EF* ejection fraction

Of note, there were no cases of aortic annulus rupture, aortic dissection, coronary ostia occlusion, or prosthesis migration.

Post procedural quantification of the AR revealed that only one patient in both groups had severe AR, in this patient the valve was implanted deep. Notably, the incidence of significant AR (more than mild AR) was significantly higher in the control group (29 % versus 12.9 %, *p* = 0.027) (Table 2).

**Table 2:** Postprocedural quantification of aortic regurgitation in both groups

	Control group	Study group	*p*-value
Post-procedural AR			
No/trace	22.6 % (*N* = 14)	21.0 % (*N* = 13)	0.9
mild	48.4 % (*N* = 30)	66.1 % (*N* = 41)	0.04
Moderate	26.4 % (*N* = 17)	12.9 % (*N* = 8)	0.03
Severe	1.6 % (*N* = 1)	0 % (*N* = 0)	0.16
High grade AR post-procedural	29.0 % (*N* = 18)	12.9 % (*N* = 8)	0.027

*AR* aortic regurgitation

## Discussion

This study searched for an acceptable method to measure the aortic annulus using 2D-TEE and its impact on the outcome after TAVI using the Medtronic CoreValve bioprosthesis.

The indications for transcatheter prosthesis size selection provided by the manufacturers are based on 2D-TEE measurements [13]. This technique was used to accurately determine the size of the aortic annulus and the excellent results of TAVI reported to date may be attributable to these complementary techniques [3, 14–16].

Two-dimensional TEE has some specific drawbacks due to the unique measurements required for TAVI. Moreover, the aortic annular diameter differs depending on whether it is measured in a coronal or sagittal plane. In the control group in this study, the longest coronal diameter in the long axis view was used for sizing. The 2D echocardiographic image of the long-axis view of the aortic valve can only show the coronet-shaped surgical annulus within the sinus as the hinge points of the visible leaflets.

Although 3-dimensional TEE lacks adequate standardization and is not yet routinely available, there is some evidence suggesting that this method is a valid alternative for more precise pre-procedural measurements in the setting of TAVI, potentially reducing the possibility of sizing errors [5]. Special software is required to reconstruct the aortic valve annulus using the virtual basal ring described by Piazza et al [17].

For instance, an oversized prosthetic valve relative to the dimensions of the patient’s aortic root can result in redundancy of leaflet tissue, thus creating folds. These folds will generate regions of compressive and tensile stresses and may alter the function or reduce the durability of the valve [18]. On the other hand, if the prosthesis is too small for the patient, the incidence of significant paravalvular regurgitation is high.

It is clear that measured dimensions by various imaging modalities employed for this purpose vary significantly The 26-mm, 29-mm and 31 mm sizes specified for the Medtronic CoreValve bioprosthesis correspond to diameters of the inflow (proximal LVOT portion) of the fully expanded stent frame in vitro [19]. The non-cylindrical nature of this bioprosthesis means that the expected dimensions at the level of the aortic annulus will vary widely, depending on the final deployed position of the device. Moreover, the heavily calcified native leaflets also take up some space at the annulus [19]. Using these data and trying to optimise sizing using 2D-TEE in the study group, the incidence of paravalvular AR was significantly less than the control group (Table 2).

Several studies [7, 20] have found that aortic annular sizing using MDCT in patients undergoing TAVI using the balloon expandable Edwards-Sapien prosthesis is the most accurate method. Nevertheless, radiation exposure, iodine injection and costs are important limitations in comparison to using the 2D-TEE. Moreover, over 50 % of patients undergoing TAVI in large studies had pre-existing chronic kidney disease and about 10 % of these patients have severe renal insufficiency [21, 22]. In these patients, MDCT is often not an option.

Most importantly, the present study demonstrates that cross-sectional measurements from 2D-TEE provide more accurate information than the long axis measurements from 2D-TEE for the performance of TAVI, with superior discrimination of post-TAVI aortic regurgitation.

## Conclusion

Sizing of the aortic valve annulus using cross-sectional 2D-TEE offers a safe and plausible method for patients undergoing TAVI using the self-expandable Medtronic CoreValve prosthesis and is significantly superior to using long axis 2D-TEE.

### Limitations

This study is a non-randomized retrospective study having its inherent limitations. On the other hand and according to the protocol of the study, we searched for a simple, cost effective method for sizing of the aortic valve annulus with acceptable results.

The resultant problem of potential “selection” bias is an inherent limitation in the design of case control studies. Trying to minimize the effect of a selection bias, we have examined the groups in two centers. One center was considered as the control center and the other is the case center. The patients were chosen consecutive at the same time period. The patient selection protocols for both centers regarding the procedure were more or less similar and according to the guidelines.

Evidence for the thresholds for sizing, as well as the optimal imaging modality for this sizing remains elusive.

## Abbreviations

TAVI: Transcatheter aortic valve implantation
2D-TEE: Two-dimensional transesophageal echocardiography
AR: Aortic regurgitation
MDCT: Multidetector computed tomography
TTE: Transthoracic echocardiography
AVA: Aortic valve area
LVOT: Left ventricular outflow tract
AV: Aortic valve
LCC: Left coronary cusp
RCC: Right coronary cusp
NCC: Non-coronary cusp
